# Quality assurance: Importance of systems and standard operating procedures

**DOI:** 10.4103/2229-3485.76288

**Published:** 2011

**Authors:** Kishu Manghani

**Affiliations:** *Proprietor-consultant, Somdev Clinical Development Associates, Mumbai, Maharashtra, India*

**Keywords:** Quality assurance, quality control, quality management, quality standards, quality systems

## Abstract

It is mandatory for sponsors of clinical trials and contract research organizations alike to establish, manage and monitor their quality control and quality assurance systems and their integral standard operating procedures and other quality documents to provide high-quality products and services to fully satisfy customer needs and expectations. Quality control and quality assurance systems together constitute the key quality systems. Quality control and quality assurance are parts of quality management. Quality control is focused on fulfilling quality requirements, whereas quality assurance is focused on providing confidence that quality requirements are fulfilled. The quality systems must be commensurate with the Company business objectives and business model. Top management commitment and its active involvement are critical in order to ensure at all times the adequacy, suitability, effectiveness and efficiency of the quality systems. Effective and efficient quality systems can promote timely registration of drugs by eliminating waste and the need for rework with overall financial and social benefits to the Company.

## INTRODUCTION

High levels of quality are essential to achieve Company business objectives. Quality, a source of competitive advantage, should remain a hallmark of Company products and services. High quality is not an added value; it is an essential basic requirement. Quality does not only relate solely to the end products and services a Company provides but also relates to the way the Company employees do their job and the work processes they follow to produce products or services. The work processes should be as efficient as possible and continually improving. Company employees constitute the most important resource for improving quality. Each employee in all organizational units is responsible for ensuring that their work processes are efficient and continually improving.

Top management should provide the training and an appropriate motivating environment to foster teamwork both within and across organizational units for employees to improve processes.

Ultimately, everyone in a Company is responsible for the quality of its products and services.

A Company in the role of a sponsor of clinical trials can best achieve its business objectives by establishing and managing robust quality systems with their integral quality documents including standard operating procedures (SOPs).

## QUALITY SYSTEMS

A quality system is defined as the organizational structure, responsibilities, processes, procedures and resources for implementing quality management. Quality management includes those aspects of the overall management function that determine and implement the Company quality policy and quality objectives. Both quality control and quality assurance are parts of quality management.

The 13^th^ principle in the International Conference on Harmonization Good Clinical Practice (ICH GCP) guideline clearly states that systems and procedures that assure the quality of every aspect of the (clinical) trial should be implemented. The sponsor is responsible for implementing and maintaining quality assurance and quality control systems with written SOPs to ensure that trials are conducted and data are generated, documented (recorded) and reported in compliance with the protocol, Good Clinical Practice (GCP) and the applicable regulatory requirements. Although a sponsor may transfer any or all of its trial-related duties and functions to a contract research organization (CRO), the ultimate responsibility for the quality and integrity of the trial data always resides with the sponsor.[1] However, the CRO is also required in its own right to always implement quality assurance and quality control. Both quality control and quality assurance systems must be commensurate with the Company business objectives and business model. The two together constitute the key quality systems.

Top management commitment and active involvement in the establishment, management and monitoring of quality systems is critical and is achieved by:[2]

- Defining and documenting a quality policy and quality objectives and ensuring that both the policy and objectives are understood and implemented by all employees at all levels;
- Ensuring that appropriate processes are implemented to fully satisfy customer needs and expectations and Company objectives;
- Defining and documenting the responsibility, authority and interrelation of key personnel managing the quality systems;
- Providing adequate resources for implementing and maintaining the quality systems;
- Conducting scheduled management reviews of the quality systems to assess their continued suitability, adequacy, effectiveness and efficiency; and
- Deciding on actions for continual quality improvement.

Quality control is focused on fulfilling quality requirements, and as related to clinical trials, it encompasses the operational techniques and activities undertaken within the quality assurance system to verify that the requirements for quality of the trial-related activities have been fulfilled.[1]

Quality assurance, on the other hand, is focused on providing confidence that quality requirements are fulfilled. As related to clinical trials, it includes all those planned and systemic actions that are established to ensure that the trial is performed and the data are generated, documented (recorded), and reported in compliance with GCP and the applicable regulatory requirements.[1]

Quality control is generally the responsibility of the operational units and quality is infused into the outputs and verified as they are being generated. Therefore, quality control is an integral part of the daily activities occurring within each operational unit.

Quality assurance is the responsibility of the quality assurance department. The mission of a quality assurance department is to provide an effective and efficient quality assurance system and counsel for the operational units. The quality assurance department must be manned by an adequate number of dedicated and adequately qualified and trained personnel with well-developed interpersonal skills. The well-developed interpersonal skills will provide the quality assurance personnel with persuasive, diplomatic, tactful and resilient qualities generally required of them. The quality assurance department must operate independently from the operational units and it must regularly perform quality review activities (self-inspection audits/internal audits) to ensure compliance within operational units with Company quality standards, good working practices [GxPs: current Good Manufacturing Practice (cGMP), Good Laboratory Practice (GLP), GCP, etc.], and local, national, regional and international legal, ethical and regulatory requirements.

The quality assurance department under the leadership of a Quality Assurance Manager will ensure the following:

- Appropriate global and affiliate-specific quality documents (Level 1: Company policies including quality policy and quality management plan; Level 2: SOPs; Level 3: working instructions; Level 4: conventions, guidelines, forms, templates, logs, tabs, and labels) are determined, developed and implemented.
- Personnel involved in clinical research and development are, and remain, properly qualified and trained for job roles for which they are made responsible. The training will include new staff induction, ongoing quality awareness training including training in applicable SOPs and other quality documents, training for changing roles within and between functional units, and training resulting from an analysis of needs including the results of audits and regulatory inspections, top management reviews and employee appraisals. Further education and additional training needs should be constantly assessed by the Company.
- All clinical research and development activities are conducted according to Company quality standards, current GxPs, and all applicable local, national, regional and international legal, ethical and regulatory requirements as defined in the quality documents, to meet with Company quality objectives and customer requirements.
- A system is put in place to track all global and affiliate-specific quality documents and to maintain an up-to-date overall inventory of all historical and effective quality documents.
- Personnel will have written job descriptions which will clearly define their roles and responsibilities, and the processes and SOPs which they have to follow.
- A system is put in place to initiate and maintain a personal file on each employee, containing his/her current curriculum vitae, job description, education and training records and personal and professional development plan.
- An auditing function, independent of the operational units and the quality control system, is created to plan, conduct, and report internal and external audits and to support and monitor their close-out via appropriate corrective actions and preventive actions (CAPA) plan.[3,4] The effectiveness of the corrective and the preventive actions must be assessed.
- A system is put in place to oversee customer audits, regulatory inspections and Company certifications/accreditations as applicable.
- A system is put in place to a) share audit and regulatory inspection findings and learning with the relevant functional units and top management, b) promote auditing-in-tandem, and cross-pollination of auditors, c) track all internal and external audits, customer audits and regulatory inspections, and d) track status of findings (open, closed or pending) made during audits and regulatory inspections.
- Liaison is maintained with functional units, affiliates, and human resources for continued personal and professional development (basic and advanced knowledge-based and skill-based training and retraining) of employees worldwide.
- Liaison is maintained with and between functional units and affiliates to promote standardization, improve communication, and to enhance efficiency of quality systems through cooperation.
- All functional units and affiliates are kept up-to-date with various established and emerging local, national, regional and international legal, ethical and regulatory standards.
- Continual quality improvement initiatives (adoption of industry best practices: determination, development, implementation and monitoring of key performance indicators; and internal and external benchmarking) are identified, implemented and monitored via the Plan–Do–Check–Act (P–D–C–A) cycle.[5,6]
- Persons responsible for the quality assurance system are available in an advisory role to employees worldwide on matters related to the quality systems, regulations in force including GxPs and regulatory compliance.

## STANDARD OPERATING PROCEDURES

Standardization is defined as an activity that gives rise to solutions for repetitive application to problems in various disciplines including science and it is aimed at achieving the optimum degree of order in a given context. Generally, the activity consists of the process of establishing (determining, formulating, and issuing) and implementing standards. Therefore, standards are the ultimate result of a standardization activity and within the context of quality systems consist of quality documents or documents related to the quality systems.

The quality documents consist of Company policies, quality management plan, SOPs, working instructions, conventions, guidelines, forms, templates, logs, tags and labels. They are established by consensus and approved by a nominated body and they provide for common and repeated use, rules, guidelines or characteristics for activities or their results with a view to promote transparency, consistency, reproducibility, interchangeability and to facilitate communication. The hierarchy and types of quality documents relevant to quality systems will depend upon Company business objectives and business model. SOPs are Level 2 quality documents and, along with other relevant quality documents, ensure the effectiveness and efficiency of quality systems.

The ICH GCP guideline defines SOPs as “detailed, written instructions to achieve uniformity of the performance of a specific function”.[1] Simply put, SOPs specify in writing, who does what and when, or the way to carry out an activity or a process. SOPs establish a systematic way of doing work and ensure that work is done consistently by all persons who are required to do the same task. SOPs must be well written in order to provide an effective control of GCP and prevent errors from occurring, thereby minimizing waste and rework. Poorly written SOPs are a source of misinformation. To be user friendly, they should be clear, unambiguous and must be written in plain language. SOPs are controlled documents and are best written by persons involved in the activity, process or function that is required to be specified or covered in the SOP. SOPs must be reviewed prior to their approval for release, for adequacy, completeness and compliance with Company standards and all applicable legal, ethical and regulatory requirements. They must be reviewed and updated as required over their life cycle and any changes made to the SOPs must be re-approved. They must bear a revision status on them and their distribution must always be documented and controlled. When obsolete SOPs are required to be retained for any purpose, they should be suitably identified to prevent unintended use. Only relevant SOPs in their current version must be available at points of use and must remain legible. SOPs are mandatory for the implementation of GCP and other GxPs, namely, cGMP and GLP, within the scope of quality systems; therefore, it is well said that without SOPs there are no GxPs: no SOPs, no quality systems, and no GxPs.

For an activity to become the topic of an SOP, it must be either subject to regulations or it must address a task important within quality systems or between quality systems and other functional units. Quality systems related SOPs should generally cover the following topics in order to capture the core quality control and quality assurance activities and processes:

- Definition, format, content, compilation, indexing, review, approval, update, distribution and archiving of quality documents;
- Definition, format, content, review, approval, update, distribution and archiving of quality management plan;
- Definition of and activities related to quality control of clinical trials and compilation of trial-specific quality control plan;
- Initiation and maintenance of personnel files including format and content of curriculum vitae, job description, training records and personal and professional development plan;
- Top management reviews of quality systems and issuance of management review reports;
- Selection and management of contract auditors;
- Format, content, compilation, review, approval, update, distribution and archiving of audit program;
- Format, content, compilation, review, approval, update, distribution and archiving of audit plan;
- Planning, conduct, reporting and close-out of risk-based internal and external audits;
- Planning, conduct, reporting and close-out of specific audits of sites, processes, systems and documents: sponsor site, third party (CRO, central clinical laboratory) site, investigator site, quality management system including SOP management, education and training and auditing, document management system including archives, data management system including information technology support, serious adverse events management system, pharmacovigilance system, medical dictionary management system, and regulatory submission documents (clinical trial reports, and clinical sections of new drug applications, marketing authorization applications, and common technical documents);
- Planning, conduct, reporting and close-out of for cause/directed audits;
- Hosting of customer audits;
- Preparation of sites for regulatory inspections;
- Coordination and management of regulatory inspections;
- Format, content, compilation, review, approval, update, distribution and archiving of CAPA plan, and assessment of its effectiveness;
- Change control to ensure that changes and the current status of quality systems related components including documents are identified; and
- Roles and responsibilities of quality assurance in handling of scientific misconduct/fraud.

## BENEFITS OF QUALITY SYSTEMS

The importance of properly established and managed quality control and quality assurance systems with their integral well-written SOPs and other quality documents for the achievement of Company business objectives cannot be ignored. They serve as a passport to success by assisting the Company to achieve high-quality processes, procedures, systems, and people, with eventual high-quality products and services and enhancement of the following:

- Customer satisfaction, and therefore, customer loyalty and repeat business and referral;
- Timely registration of drugs by eliminating waste and the need for rework;
- Operational results such as revenue, profitability, market share and export opportunities;
- Alignment of processes with achievement of better results;
- Understanding and motivation of employees toward the Company quality policy and business objectives, as well as participation in continual quality improvement initiatives; and
- Confidence of interested parties in the effectiveness and efficiency of the Company as demonstrated by the financial and social gains from Company performance and reputation.
